# The self-assessment of critical thinking disposition and the needs for training: a cross-sectional survey of clinical nurses

**DOI:** 10.3389/fmed.2025.1653991

**Published:** 2025-08-20

**Authors:** Kailun Gao, Xiaotong Zhong, Yingying Zhang, Min Wang, Ling Chen, Wenzhi Cai, Wei Ren

**Affiliations:** ^1^Department of Nursing, Shenzhen Hospital, Southern Medical University, Shenzhen, China; ^2^School of Nursing, Southern Medical University, Guangzhou, China

**Keywords:** critical thinking disposition, training needs assessment, clinical nurses, nurse education, cross-sectional study

## Abstract

**Background:**

Critical thinking is essential for nurses’ clinical decision-making and the delivery of safe, high-quality care, and is shaped by their critical thinking disposition. However, nurses demonstrate negative critical thinking dispositions, with limited evidence available on influencing factors and training needs.

**Objective:**

This study aims to investigate nurses’ critical thinking dispositions, identify influencing factors, and explore their relationship with training needs.

**Methods:**

A cross-sectional study was conducted in three tertiary Grade A hospitals from January to February 2025. Nurses were asked to complete the Chinese version of the critical thinking disposition inventory and a self-designed training needs questionnaire. Descriptive statistics, correlation analysis, and multiple regression analysis were performed.

**Results:**

A total of 370 clinical nurses participated in this study. The average critical thinking disposition score was 281.58 ± 36.68 and training needs score was 90.94 ± 12.01. Age, working experience, professional level, specialist nurse, position, department, and health status significantly influenced critical thinking disposition (all *p* < 0.05). Multiple linear regression analysis showed health status and position being the most significant contributors (*R*^2^ = 0.128, *p* < 0.001). Notably, a significant positive correlation was observed between nurses’ critical thinking disposition and their training needs (*r* = 0.358, *p* < 0.01).

**Conclusion:**

These findings highlight the necessity of aligning education with individual needs. By implementing tailored training programs, nurses can be better supported the development of critical thinking, thereby fostering safer and higher-quality clinical practice.

## 1 Introduction

Ensuring high-quality nursing care is a global health priority and a fundamental component of patient safety, as emphasized by the global health organisations (1). As the most directly engaged groups of healthcare professionals, nurses are confronted with growing healthcare demands resulting from an aging population, the rising prevalence of chronic, and rapid advances in medical technology (2). To meet these demands, nurses must possess not only adequate institutional support and staffing, but also the development of core competencies that underpin clinical decision-making, effective communication, and improved patient outcomes (3, 4). Among the core competencies required for high-quality nursing care, critical thinking is widely recognized as fundamental to ensuring safe, efficient, and high-quality care (5, 6).

Critical thinking in nursing is the ability to purposefully analyze information, reflect on clinical cues, make evidence-informed decisions, and act proactively in unpredictable situations (7, 8). Its relevance spans all phases of the nursing process. For example, during patient assessment, critical thinking helps nurses distinguish between relevant and irrelevant data, connect subtle signs with potential complications, and anticipate deterioration before it becomes clinically obvious (9). In the planning and implementation phases, it supports the selection of individualized interventions that align with dynamic patient conditions (10), while in the evaluation phase, it enables reflective appraisal of outcomes and adjustments to care strategies (11). Therefore, insufficient critical thinking skills may lead to delayed nursing interventions and heightened risks to patient safety.

Beyond its impact on nursing processes, critical thinking also enhances nurses’ self-confidence, job satisfaction, and resilience, while fostering professional growth through greater initiative and accountability (12–14). However, despite its recognized importance, nurses in countries such as China, Vietnam, and Spain often demonstrate a relatively negative disposition toward critical thinking (15–17). This may stem from rigid clinical hierarchies, task-oriented work cultures, and educational models that emphasize technical skills over independent thinking (18, 19). In China specifically, deference to authority rooted in Confucian values may further hinder the cultivation of critical thinking among junior nurses (20).

Although efforts have been made to incorporate critical thinking into nurse education, there remains a lack of empirical evidence regarding the current disposition of nurses, the factors that influence it, and how these factors relate to training needs (21, 22). Without such evidence, attempts to strengthen critical thinking through education and training may be ineffective or poorly targeted. In this context, Knowles’ theory of adult learning provides a valuable perspective for understanding the potential relationship between critical thinking disposition and training needs (23). Specifically, nurses with a more positive disposition toward critical thinking may be more attuned to their own cognitive development gaps and, consequently, exhibit a greater demand for training opportunities.

The purpose of the current study is to investigate the critical thinking dispositions of nurses, examine the influencing factors, and explore their relationship with training needs. The findings are intended to provide a scientific basis for nursing administrators and educators to enhance nurses’ critical thinking and ultimately enhance care quality and patient safety.

## 2 Methods

### 2.1 Design

This study employed a cross-sectional design, adhering to the Strengthening the Reporting of Observational Studies in Epidemiology (STROBE) reporting guidelines for observational research (24).

### 2.2 Setting and participants

This study employed convenience sampling to survey nurses working in three tertiary Grade A hospitals in Guangdong province in southeast China from January 2025 to February 2025. The inclusion criteria were as follows: (1) possession of a valid nursing license; (2) current employment as a registered nurse in a healthcare institution; and (3) willingness to provide informed consent and participate voluntarily in the study. The exclusion criterion was nurses who were on leave for more than 3 months during the survey period.

The sample size was calculated via the multiple linear regression formula N > 50 + 8*m (25), where m represents the number of independent variables. This study included ten sociodemographic variables, seven dimensions of the Chinese version of the critical thinking disposition inventory (CTDI-CV), and 5 dimensions of training needs as independent variables. On this basis, a minimum of 226 participants (50 + 22*8) were needed. Accounting for a 20% attrition rate, the target sample size was increased to 283 participants. Ultimately, a total of 370 nurses participated in the study.

### 2.3 Instruments

The questionnaire consists of three parts:

Demographic variables, including age, working experience, gender, marital status, professional level, educational background, specialist nurse, position, department, and health status.

The Chinese version of the Critical Thinking Disposition Inventory (CTDI-CV) (26) was adapted by Peng et al. from the California Critical Thinking Disposition Inventory (CCTDI) (27). The scale comprises seven dimensions: truth-seeking, open-mindedness, analyticity, systematicity, self-confidence, inquisitiveness, and maturity. Each dimension consists of ten items, totaling 70 items. A 6-point Likert scale (1–6) was used, with 30 positively scored items (higher agreement indicating higher scores) and 40 negatively scored items (higher disagreement indicating higher scores). The total score ranges from 70 to 420, with a score greater than 280 points indicating a positive critical thinking disposition, a score between 211 and 279 points suggesting ambivalence, and a score below 210 points reflecting a negative. The scale’s Cronbach’s *α* coefficient is 0.90 (26). In this study, the Cronbach’s α coefficient was 0.91.

Critical thinking training needs questionnaire based on a comprehensive literature review and key concepts related to critical thinking and training needs assessment (28, 29). The instrument was refined through expert panel discussions and is structured around the five core dimensions of the nursing process: assessment (4 items), diagnosis (4 items), planning (4 items), implementation (6 items), and evaluation (2 items), a total of 20 items. Responses are recorded on a five-point Likert scale, ranging from “very unnecessary” to “very necessary,” with higher scores indicating a stronger perceived need for critical thinking training. A small-scale pilot study was conducted before formal distribution, and the instrument demonstrated high internal consistency, with a Cronbach’s *α* coefficient of 0.96.

### 2.4 Data collection

In this study, the questionnaire was administered via the Questionnaire Star platform. Before distribution, approval was obtained from the nursing department director to contact nursing managers across various departments. Two researchers, after receiving uniform training, administered the questionnaires to nurses in their respective departments. All participants provided written informed consent. All items in the questionnaire are needed, and only one response per IP address is permitted to prevent duplicate submissions. The collected data were independently reviewed by two researchers and questionnaires with short completion times (≤120 s) or illogical responses were excluded.

### 2.5 Data analysis

Data were entered into Excel 2019 and analyzed using IBM SPSS version 26.0. Descriptive statistics were first employed to determine the distributions of variables. Univariate analysis was then conducted to compare critical thinking disposition scores across the independent variable groups. Subsequently, variables demonstrating significant associations were included in multiple linear regression. Additionally, a Spearman’s correlation analysis was performed to assess the relationship with training needs. A *p*-value of < 0.05 was considered statistically significant (two-sided).

### 2.6 Ethical approval

Ethical approval was received from the Ethics Committees of Shenzhen Hospital, Southern Medical University (No. NYSZYYEC2024K050R001). The data obtained in interviews were anonymized and safely kept to protect the privacy of study subjects.

## 3 Results

### 3.1 Nurses’ critical thinking disposition scores and training needs scores

A total of 370 responses were collected, and 344 valid questionnaires were retained after data screening, yielding a valid response rate of 92.98%. The total score for the surveyed nurses’ critical thinking disposition was 281.58 ± 36.68, with an average item score of 4.02 ± 0.52. Additional data are described in Table 1. The total score for critical thinking training needs was 90.94 ± 12.01. Among the subdimensions, nursing implementation received the highest score with a score rate of 91.67% (mean = 27.50, SD = 3.81), followed by nursing diagnosis (90.90%, mean = 18.18, SD = 2.60), nursing evaluation (90.80%, mean = 9.08, SD = 1.36), nursing assessment (90.50%, mean = 18.10, SD = 2.65), and nursing plan (90.35%, mean = 18.07, SD = 2.61).

**Table 1:** Nurses’ critical thinking disposition scores (*N* = 344).

CTDI-CV scores	Mean ± SD	Critical thinking dispositions *N* (%)		
		Negative	Ambivalence	Positive
Total scores	281.58 ± 36.68	2 (0.58)	168 (48.84)	174 (50.58)
Subdimensions				
Truth-seeking	35.41 ± 9.81	94 (27.33)	135 (39.24)	115 (33.43)
Open-mindedness	39.38 ± 7.14	40 (11.63)	140 (40.70)	164 (47.67)
Analyticity	42.97 ± 5.27	2 (0.58)	94 (27.33)	248 (72.09)
Systematicity	40.35 ± 7.21	21 (6.10)	139 (40.41)	184 (53.49)
Self-confidence	42.39 ± 7.26	15 (4.36)	113 (32.85)	216 (62.79)
Inquisitiveness	43.68 ± 7.46	15 (4.36)	85 (24.71)	244 (70.93)
Maturity	37.39 ± 9.95	88 (25.58)	110 (31.98)	146 (42.44)

**Figure 1:**
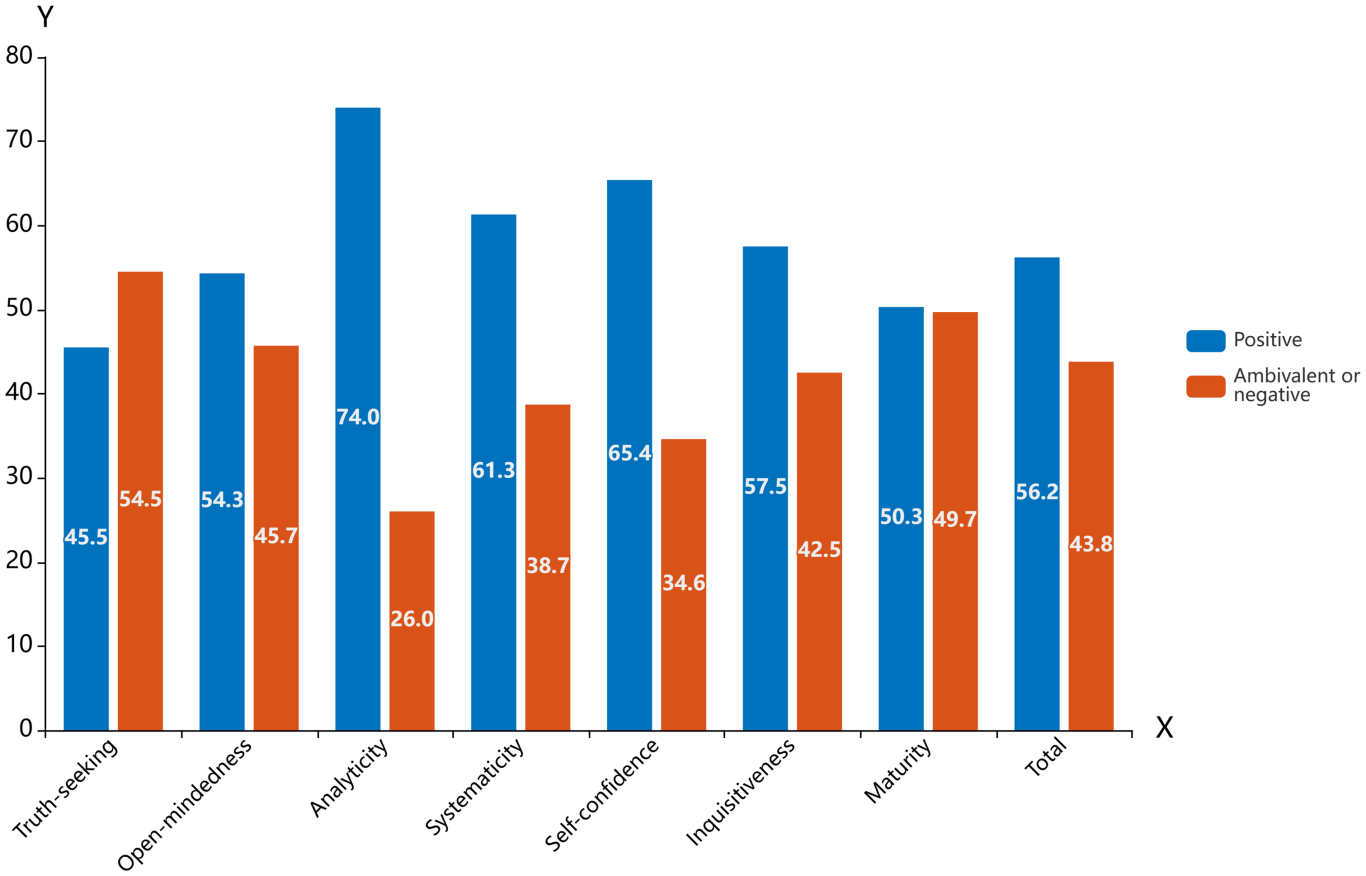
The critical thinking dispositions of nurses (*N* = 344) are presented, illustrating the percentages of respondents (*Y*-axis) with positive, ambivalent or negative dispositions across each sub-scale and the total score (*X*-axis).

When the total critical thinking disposition score was divided into two parts: positive disposition and ambivalent or negative disposition, half of the respondents had a positive disposition (i.e., a score of 280 points or above), 170 nurses (48.84%) showed ambivalent or negative disposition, while no respondents demonstrated a strongly negative disposition. Overall, the findings suggest that the nurses’ critical thinking abilities were at a moderate level (Figure 1).

### 3.2 Univariate analysis of nurses’ critical thinking disposition scores

The 344 nurses surveyed had a mean age of 34.72 ± 7.60 years and an average work experience of 12.68 ± 8.50 years. A one-way analysis of variance was conducted to assess the relationship between nurses’ general characteristics and their critical thinking disposition scores. The results indicated statistically significant differences in critical thinking disposition scores based on factors such as age, working experience, professional level, specialist nurse, position, department, and health status. General characteristics and univariate analysis are presented in Table 2.

**Table 2:** Univariate analysis of nurses’ critical thinking (*N* = 344).

Variables	*N* (%)	*t/F*	*p*
Age (years)		6.940	**0.001**
30	121 (35.2)		
31–40	142 (41.3)		
41	81 (23.5)		
Working experience (years)		7.044	**0.001**
10	157 (45.6)		
11–20	128 (37.2)		
21	59 (17.2)		
Gender		0.968	0.326
Male	22 (6.4)		
Female	322 (93.6)		
Marital status		1.303	0.273
Single	95 (27.6)		
Married	240 (69.8)		
Others	9 (2.6)		
Professional level		2.7	**0.046**
N0	25 (7.3)		
N1	48 (14.0)		
N2	115 (33.4)		
N3 and above	156 (45.3)		
Educational background		1.076	0.342
College degree and below	29 (8.4)		
Bachelor degree	286 (83.1)		
Postgraduate degree	29 (8.4)		
Specialist nurse		2.208	**0.028**
Yes	143 (41.6)		
No	201 (58.4)		
Position		12.590	**<0.001**
Nurse	287 (83.4)		
Nurse Manager	53 (15.4)		
(Deputy) Director of Nursing	4 (1.2)		
Department		4.07	**<0.001**
Internal medicine	61 (17.7)		
Surgery	97 (28.2)		
Gynecology	19 (5.5)		
Pediatrics	15 (4.4)		
Intensive care medicine	41 (11.9)		
Operating room	5 (1.5)		
Nursing department	12 (3.5)		
Other	94 (27.3)		
Health status		5.79	**0.003**
Good	271 (78.8)		
General	65 (18.9)		
Poor	8 (2.3)		

Bold values indicate statistical significance at *p* < 0.05.

### 3.3 Multifactorial analysis of nurses’ critical thinking disposition scores

A multiple linear regression analysis was conducted using the variables that showed significant differences in the univariate analysis as independent variables, with the nurses’ critical thinking disposition scores as the dependent variable (actual scores included). The coding scheme for the independent variables is presented in Supplementary Table 1. The results of the multiple linear regression analysis show that the adjusted *R*^2^ = 12.8% (*F* = 4.558, *p* < 0.001), with nurses’ health status and position being the important influencing factors of their critical thinking disposition scores (Table 3).

**Table 3:** Multiple linear regression model for nurses’ critical thinking disposition scores (*N* = 344).

Variables	Unstandardized coefficients		Standardized coefficients	*t*	*p*	Tolerance	VIF
	*β*	Standardized error	Beta				
Constant	244.834	13.388		18.288	<0.001		
Age (years)	5.215	4.908	0.108	1.063	0.289	0.247	4.051
Working experience (years)	−3.467	5.048	−0.70	−0.687	0.493	0.245	4.085
Professional level	0.406	2.537	0.011	0.160	0.873	0.551	1.815
Specialist nurse	4.468	3.976	0.060	1.124	0.262	0.888	1.126
Health status	11.804	3.980	0.153	2.966	0.003	0.951	1.052
Position							
Nurse Manager	14.994	5.927	0.148	2.530	0.012	0.745	1.342
(Deputy)Director of Nursing	59.217	21.346	0.173	2.774	0.006	0.651	1.535
Department							
Surgery	−8.052	5.651	−0.099	−1.425	0.155	0.528	1.895
Gynecology	−0.446	9.088	−0.003	−0.049	0.961	0.792	1.263
Pediatrics	23.785	9.943	0.133	2.392	0.017	0.827	1.209
Intensive care medicine	−21.831	7.066	−0.193	−3.090	0.002	0.651	1.536
Operating room	4.309	16.070	0.014	0.268	0.789	0.922	1.084
Nursing department	−23.478	13.003	−0.118	−1.806	0.072	0.599	1.669
Other	−4.781	5.726	−0.058	−0.835	0.404	0.524	1.909

*R* = 0.405, *R*^2^ = 0.164, Adjusted *R*^2^ = 0.128, *F* = 4.558.

### 3.4 Correlation between nurses’ critical thinking disposition and training needs scores

**Figure 2:**
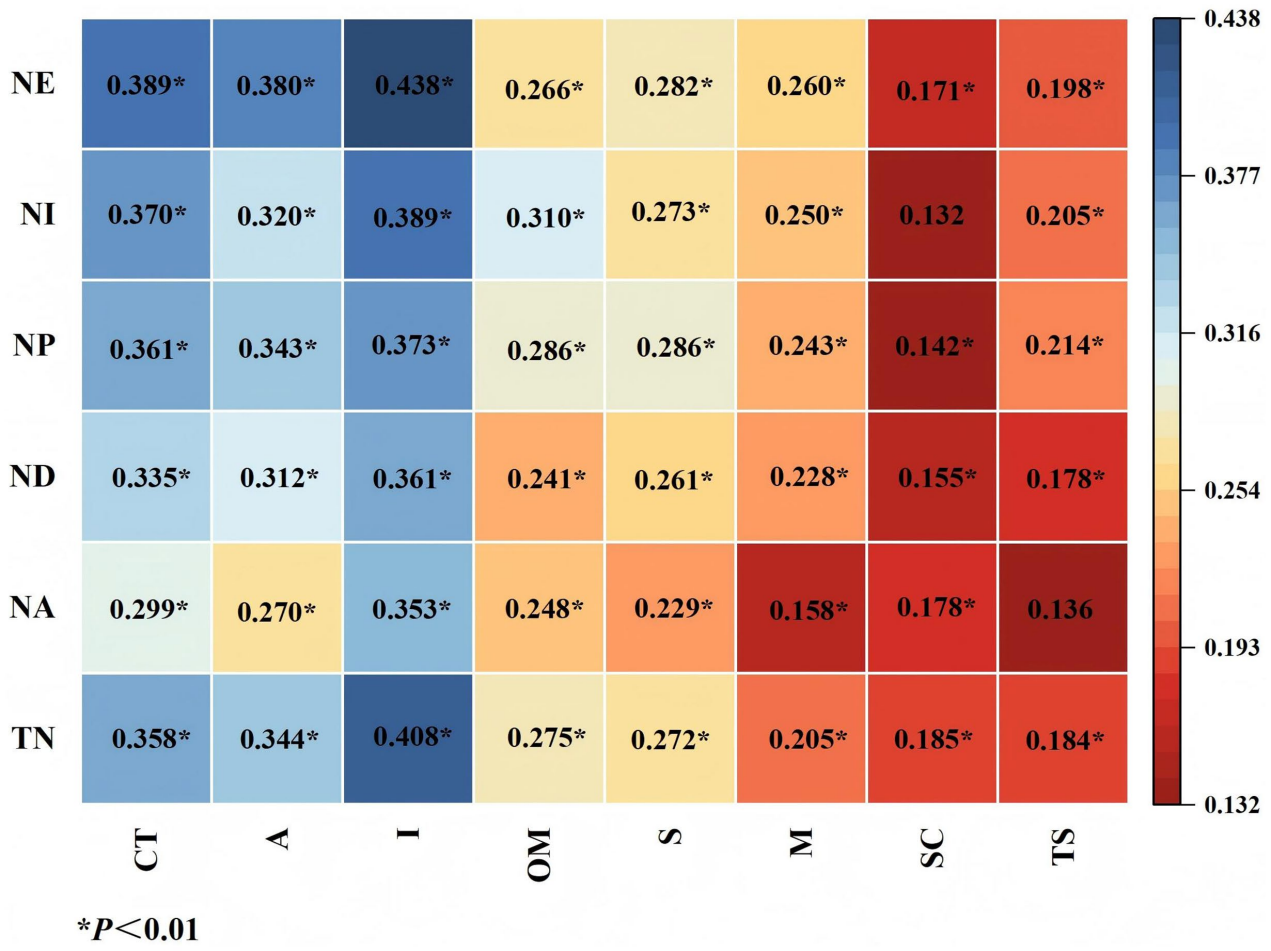
Correlation heatmap of critical thinking disposition and training needs scores (*p* < 0.01). CT, CTDI-CV scores, including A, analyticity; I, inquisitiveness; OM, open-mindedness; S, systematicity; M, maturity, SC, self-confidence; and TS, truth-seeking; TN, training needs scores, including NA, nursing assessment; ND, nursing diagnosis; NP, nursing plan; NI, nursing implementation; and NE, nursing evaluation.

To explore the relationship between nurses’ critical thinking disposition and their training needs, a Spearman correlation analysis was performed. As shown in Figure 2, several significant correlations were observed. The heatmap displays positive correlations between critical thinking disposition and training needs, with correlation coefficients ranging from 0.132 to 0.438. All correlations were statistically significant (*p* < 0.05). The color gradient from red to blue represents the strength of the positive correlations. Darker blue shades correspond to stronger positive correlations, while darker red shades indicate weaker positive correlations. The values suggest that the relationships between the variables are generally weak to moderate in strength. These findings suggest that higher critical thinking abilities are generally associated with greater perceived training needs among nurses.

## 4 Discussion

Critical thinking is essential for nurses to provide safe and effective care and fosters a more proactive approach to nursing practice (30). This study showed that nurses’ critical thinking disposition score was 281.58 ± 36.68, indicating an overall positive disposition. Factors such as age, working experience, professional level, specialist nurse, position, department, and health status may be associated with critical thinking disposition. Multiple linear regression analysis showed health status and position being the most significant contributors. Notably, the average training needs score was 90.94 ± 12.01, which showed a positive correlation with their critical thinking disposition (*r* = 0.358, *p* < 0.01).

The result of this study has been found to be consistent with previous studies that utilized the CTDI-CV to measure the critical thinking disposition of nursing students and clinical nurses (15, 17). In addition, similar conclusions have been drawn by studies using different instruments to assess nurses’ critical thinking disposition. Nguyen et al. in Vietnam utilized an 11-item Critical Thinking Disposition Scale and Barry et al. in Iran employed Ricketts’ Critical Thinking Disposition Assessment Scale, both finding that nurses demonstrated a moderate level of critical thinking (31, 32). However, compared with data from developed countries, the critical thinking disposition scores in this study remain relatively low (33, 34). This discrepancy may be influenced by factors such as educational models and the cultural context. The Chinese education system is deeply influenced by Confucian philosophy, which emphasizes respect for teachers and deference to authority (31). To some extent, this cultural tradition may impact the development of critical thinking.

Furthermore, among the seven sub-dimensions of the CTDI-CV, nurses scored higher in analyticity, self-confidence, and inquisitiveness. This finding is consistent with the study by Sun et al. (15). These aspects may be related to the characteristics of clinical nursing practice (35), which are likely reinforced through clinical decision-making and continuous patient monitoring. Therefore, nurse managers and educators can foster a supportive clinical learning environment that encourages reflective thinking, critical questioning, and independent problem-solving, such as complex nursing case discussions.

On the other hand, the lowest average score was observed on the subscale of truth-seeking, with the majority of participants’ scores reflecting a negative tendency towards this dimension. This finding is consistent with existing literature indicating that truth-seeking is a challenging dimension for nurses (36), potentially reflecting educational and cultural influences. In traditional Chinese nursing education, learning outcomes are primarily evaluated through standardized testing (15). This may limit nurses’ ability to think independently and actively seek truth in clinical practice, thereby impeding the development of critical thinking (37). To mitigate the influence of traditional educational approaches, nurse managers and educators are encouraged to foster independent thinking by implementing structured and reflective learning interventions. Strategies such as interdisciplinary training programs, evidence-based practice workshops, and reflective practice sessions can create a psychologically safe learning environment that empowers nurses to question assumptions, articulate clinical reasoning, and make evidence-informed decisions.

It is noteworthy that nearly 49% of nurses in this study were categorized as having an ambivalent critical thinking disposition. From the perspective of adult learning theory (23), it may retain intrinsic motivation for development but may lack sufficient support or external stimuli to actively cultivate their critical thinking. Therefore, this subgroup represents an important target for future educational interventions. Tailored strategies, such as reflective practice, structured mentorship programs, and active participation in interdisciplinary discussions, could be particularly effective in fostering the critical thinking abilities of these nurses.

Although previous studies reported no association between critical thinking levels and sociodemographic characteristics (8), the present study found that critical thinking disposition was potentially linked to age, working experience, professional level, specialist nurse, position, department, and health status. To gain a more comprehensive understanding of these associations, the study conducted a multiple regression analysis and found that health status and position were important influencing factors. Nurses in good health can possess greater mental energy reserves, which may facilitate more effective problem analysis and decision-making (38). In addition, Nurses in different positions exhibit varying critical thinking abilities due to differences in responsibilities and experience (13). These findings suggest that nursing administrators can enhance the critical thinking of their teams by optimizing shift schedules and providing professional development opportunities. However, it is important to note that the adjusted *R^2^* of the regression model was relatively low (0.128), indicating that the variables included in the model explained only a limited proportion of the variance in training needs. This result suggests that other important factors such as workplace culture, leadership style, and institutional training policies may have influenced the outcomes but were not captured in the current model.

Moreover, this study also assessed nurses’ training needs in critical thinking, which shows that the nurses’ critical thinking training needs score is 90.94 ± 12.01, exhibiting a strong demand. Among the sub-dimensions, the highest need is observed in the nursing implementation dimension. This may be attributed to the modern healthcare emphasis on patient-centered care, in which nurses must respond to needs during nursing implementation based on critical thinking (39). To address this, nursing managers should prioritize strengthening critical thinking in nursing implementation through a diverse range of interventions, such as scenario-based simulation training, case-based learning, reflective practice sessions, and interprofessional discussion groups.

Notably, this study also found a positive correlation between nurses’ critical thinking disposition and their training needs (*r* = 0.358, *p* < 0.01). Nurses with positive critical thinking disposition may be more sensitive to knowledge gaps in their clinical decision-making, motivating them to actively seek training to address these gaps. This aligns with the concept of “self-directed learning” in adult learning theory (23). This study also found that the dimensions of analyticity (*r* = 0.344, *p* < 0.01) and inquisitiveness (*r* = 0.408 *p* < 0.01) were most strongly correlated with nurses’ training needs, indicating that these cognitive traits play important roles in shaping training needs. Nurses with stronger analytical skills are better equipped to assess complex needs, while inquisitiveness motivates them to proactively explore new knowledge and skills. These findings highlight the differentiated impact of cognitive traits on learning needs and provide a theoretical foundation for the development of personalized nursing training programs. To facilitate actual behavior change among nurses, training programs should integrate training into real-world clinical contexts, provide ongoing feedback, and create supportive institutional environments that encourage application of new skills. These approaches, grounded in adult learning theory and the Kirkpatrick evaluation model, can bridge the gap between theoretical knowledge and clinical practice, fostering sustained improvements in critical thinking (23, 40).

## 5 Limitations of the study

This study employed a cross-sectional design and recruited nurses from three tertiary Grade A hospitals in southeast China using a convenience sampling method. Due to the non-random sampling method and potential differences among nurses from various hospital levels, the generalizability of the findings may be limited and may not fully represent the broader nursing population. Additionally, the use of self-reported questionnaires introduces the possibility of subjectivity and social desirability bias, which could affect the accuracy of the reported perceptions and behaviors. Although the training needs questionnaire demonstrated good internal consistency and expert review, it has not undergone formal external validation, which may impact its validity. Future studies should expand the sample size and adopt random sampling methods after conducting external validation to increase the representativeness and generalizability of the findings. Furthermore, to mitigate the influence of social desirability bias, future studies could consider applying indirect questioning techniques to encourage more authentic responses. Then, incorporating longitudinal research designs could enable the tracking of nurses’ critical thinking development and the effectiveness of training.

## 6 Conclusion

This study found that nurses generally exhibited a positive disposition toward critical thinking, which was significantly positively correlated with their training needs. These findings highlight the necessity of aligning education with individual needs. It is recommended that nursing training programs incorporate evidence-based and learner-centered approaches tailored to critical thinking characteristics and training needs. By facilitating its integration into daily clinical decision-making, strategies such as blended learning, structured mentorship, reflective practice, and interprofessional case discussions can promote sustained behavioral change and ultimately improve nursing care quality.

## Data Availability

The raw data supporting the conclusions of this article will be made available by the authors, without undue reservation.
