# Supplementary material for: The self-assessment of critical thinking disposition and the needs for training: a cross-sectional survey of clinical nurses
Source: Front Med (Lausanne). 2025 Aug 20;12:1653991. doi: 10.3389/fmed.2025.1653991 (PMC12406705; doi:10.3389/fmed.2025.1653991)
Supplement: Supplementary file 1 [file Table_1.pdf]

Supplementary Table 1

Assignment table of argument variables

Variable	Name		Assignment
**Age**	Z1		- 30=1, 31 - 40=2, 41 - =3
**Working experience**	Z2		- 10=1, 11 - 20=2, 21 - =3
**Professional level**	Z3		N0=1, N1=2, N2=3, N3 and above=4
**Specialist nurse**	Z4		No=1, Yes=2
**Health status**	Z5		Good=1, General=2, Poor=3
**Position**	Z6	Nurse=0	Nurse Manager（Z61=1, Z62=0)
			(Deputy)Director of Nursing (Z61=0, Z62=1)
**Department**	Z7	Internal medicine=0	Surgery (Z71=1, Z72=0, Z73=0, Z74=0, Z75=0, Z76=0, Z77=0)
			Gynecology (Z71=0, Z72=1, Z73=0, Z74=0, Z75=0, Z76=0, Z77=0)
			Pediatrics (Z71=0, Z72=0, Z73=1, Z74=0, Z75=0, Z76=0, Z77=0)
			Intensive care medicine (Z71=0, Z72=0, Z73=0, Z74=1, Z75=0, Z76=0, Z77=0)
			Operating room (Z71=0, Z72=0, Z73=0, Z74=0, Z75=1, Z76=0, Z77=0)
			Nursing department (Z71=0, Z72=0, Z73=0, Z74=0, Z75=0, Z76=1, Z77=0)
			Other (Z71=0, Z72=0, Z73=0, Z74=0, Z75=0, Z76=0, Z77=1)
